# Amniotic Fluid: A Perspective on Promising Advances in the Prevention and Treatment of Necrotizing Enterocolitis

**DOI:** 10.3389/fped.2022.859805

**Published:** 2022-03-14

**Authors:** Rimke Romee de Kroon, Tessa de Baat, Stefania Senger, Mirjam Maria van Weissenbruch

**Affiliations:** ^1^Department of Neonatology, Amsterdam University Medical Center, Amsterdam, Netherlands; ^2^Department of Mucosal Immunology and Biology Research Center, Massachusetts General Hospital for Children, Boston, MA, United States

**Keywords:** neonatology, pediatric, gastro-enterology, amniotic fluid, necrotizing enterocolitis

## Abstract

Necrotizing enterocolitis (NEC) is a common and potentially fatal disease that typically affects preterm (PIs) and very low birth weight infants (VLBWIs). Although NEC has been extensively studied, the current therapeutic approaches are unsatisfactory. Due to the similarities in the composition between human amniotic fluid (AF) and human breast milk (BM), which plays a protective role in the development of NEC in PIs and VLBWIs, it has been postulated that AF has similar effects on the outcome of NEC and potential therapeutic implications. AF has been long used for its diagnostic purposes and is often discarded after birth as “biological waste”. However, researchers have started to elucidate its therapeutic potential. Experimental studies in animal models have shown that diseases of various organ systems can possibly benefit from AF-based therapy. Hence, we have identified three approaches which show promising results for future clinical application in the prevention and/or treatment of NEC: (1) administration of processed AF (PAF) isolated from donor mothers, (2) administration of AF stem cells (AFSCs), and (3) administration of simulated AF (SAF) formulated to mimic the composition of physiological AF. We have highlighted the most important aspects that should be taken into account to guide further research on the clinical application of AF-based therapy. We hope that this review can provide a framework to identify the challenges of AF-based therapy and help to design future studies to better evaluate AF-based approaches for the treatment and/or prevention of NEC in PIs and VLBWIs.

## Introduction

Necrotizing enterocolitis (NEC) is a common and potentially fatal disease that typically affects preterm infants (PIs) and very low birth weight infants (VLBWIs). NEC is an inflammatory disease of the intestines, which ranges from mucosal injury to bowel necrosis and perforation. Although NEC is extensively studied, the current therapeutic approaches are unsatisfactory and mortality and long-term morbidity remain high (1). Breast milk (BM) feeding has a protective role on the development of NEC and is associated with reduced NEC incidence when compared to formula feeding (2). Since factors that are present in BM are also found in amniotic fluid (AF), it has been hypothesized that AF has similar protective effects (3). AF is widely used for its diagnostic purposes and is often considered as “biological waste”. However, researchers have started to elucidate the therapeutic potential of AF in *in vitro* and *in vivo* studies in animal models (4, 5). Our intent was to identify relevant research on AF, its impact on the gastrointestinal tract (GIT) and to investigate the potential therapeutic application of AF on the development of NEC. We aim to define a theoretical framework to guide future research on clinical application of AF-based therapy against NEC.

## The Pathogenesis of Necrotizing Enterocolitis

The pathogenesis of NEC is understood as a complex and multifactorial process, in which the immature intestines and microbial dysbiosis play a pivotal role (1). PIs are particularly susceptible to developing NEC by facilitating an exaggerated inflammatory response to colonizing bacteria in the premature gut. The abnormal gut microbiota of PIs has been linked to NEC pathogenesis (6). Moreover, Toll-like receptor 4 (TLR4) signaling is proposed to play a role. TLR4 is expressed at higher levels on the intestinal epithelium in PIs and leads to increased apoptosis of enterocytes, hampered intestinal mucosal healing and the production of pro-inflammatory cytokines (6, 7). BM, and potentially AF, provides important protective mechanisms to diminish the inflammatory response in the neonatal intestines and reduce the risk of NEC.

## Amniotic Fluid is a Complex and Dynamic Biological Fluid

The fetus develops in the amniotic sac. Initially, AF is synthesized from maternal plasma and absorbed through the fetal skin. Subsequently, the fetus contributes to the production of AF by urination, through the trans-membranous pathway and other pathways of secretion. After keratinization of the skin, the fetus takes up AF through the GIT. Until the 20th week of gestation, the content of AF is similar to that of fetal plasma. Following skin keratinization, the content changes (8). Water generally accounts for 98%, while the remainder consists of soluble components: minerals, proteins, carbohydrates, lipids, steroids and hormones (9). AF also contains exfoliated skin, respiratory tract, urinary tract and GIT cells as well as immune cells. Furthermore, AF consists of a heterogenous pool of AF-specific cells, a small percentage of which are AF stem cells (AFSCs) (10). AF functions as a protective fluid for the fetus by protecting against physical trauma, supporting the umbilical cord and lubricating the fetal skin. AF also helps to protect against fetal infection through immune cells, microbial peptides and enzymes. Lastly, the majority of nutrition is provided by the placenta but AF is also a key source of nutrition (8, 9).

## Amniotic Fluid Contributes to the Development of the Gastro-Intestinal Tract in Utero

The formation of the primitive gut initiates during organogenesis. Following this, the rudimentary gut tube forms and the fetus starts contributing to AF. During late gestation, the crypts and villi mature, resulting in a gut that is ready for extra-uterine life (11). Exposure to AF plays an important role in the development of the fetal intestines. Studies in pig fetuses that had undergone esophageal ligation, which limits the ingestion of AF, demonstrated that ligated pig fetuses have diminished GIT development (12). In line with this, multiple trophic factors (TFs) in AF benefit fetal intestinal development (13). Exposure to growth factors (GFs), including fibroblast GF (FGF), epidermal GF (EGF), hepatocyte GF (HGF), insulin-like GF (IGF)-1, IGF-2 and transforming GF (TGF)-α, promoted fetal intestinal growth, similar to BM, in an *in vitro* model (14). After birth, exposure to AF comes to a halt and development of the GIT continues in the neonatal phase, when the GIT is exposed to microbes. Feeding with BM contributes to mucosal differentiation and further intestinal development (15).

## The Diagnostic Application of Amniotic Fluid

Evaluation of AF has taken a prominent place in prenatal diagnostics. AF can be obtained during pregnancy through amniocentesis or chorionic villus sampling. Cytogenic assessment of isolated AF is routinely used to diagnose chromosomal disorders (16). More recently developed AF-based tests aim to optimize monitoring of fetal and maternal health, for example through analysis of cell-free DNA and whole exome sequencing (17). AF volume is also critical for fetal growth and development. Abnormalities in its volume are associated with pathologies. The assessment of AF volume can thus improve pregnancy outcomes (18).

## Amniotic Fluid-Based Therapy in Neonates With Necrotizing Enterocolitis

**Figure 1:**
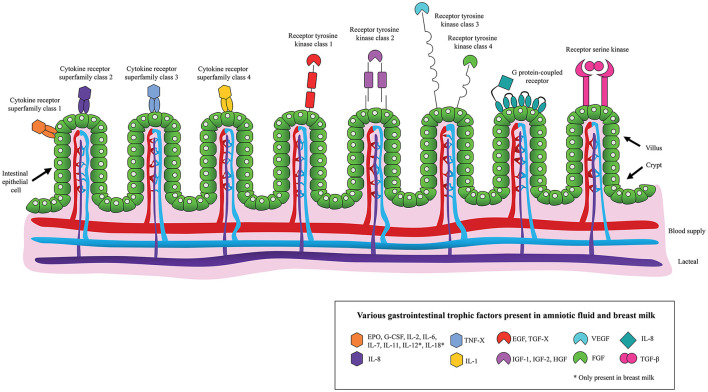
Schematic overview of the binding of various gastrointestinal trophic factors, present in amniotic fluid and/or breast milk, to the neonatal intestinal epithelium.

In addition to using AF for diagnostic purposes, studies on its therapeutic use are increasing. Considering that AF promotes the development of a healthy GIT and has a similar composition to BM, including the presence of EGF, TGF-β, IGF-1, IGF-2, interleukins (ILs), lactoferrin and immunoglobulins (Figure 1), it has been hypothesized that AF and its derivatives may limit the development of NEC (13, 19). Here, we discuss key studies on this topic. To assess the risk of bias in the animal studies that were employed the SYRCLE's risk of bias tool was used (Supplementary Table 1) (20).

### Postnatal Enteral Amniotic Fluid Administration

A new area of research interest focuses on utilizing processed AF (PAF) (4, 5). Human AF (HAF) is collected through amniocentesis or during Cesarean section. HAF is centrifuged and the supernatant is processed using filtration technology to sterilize and eliminate cellular debris. AF contains a variety of defense proteins, cytokines, antimicrobial peptides and other antibacterial components, which are maintained after processing (21, 22). In PIs, the exposure of the fetal intestines to AF is terminated abruptly. It has been argued that sudden loss of exposure of AF might increase the risk of developing NEC (23). Hence, postnatal enteral administration of PAF is hypothesized to reduce NEC development and has been investigated in established animal models (Supplementary Table 2).

TLR4 signaling, which is dampened by AF *in utero*, is inhibited in the gut of fetal mice (tested on day 18.5 of gestation, term gestation is 19–21 days) treated with lipopolysaccharide (LPS) and AF compared to fetal mice stimulated with LPS and a saline solution. Exposure to AF resulted in reduced NEC severity and decreased levels of inducible nitric oxide synthase expression. The latter reflects reduced TLR4 activation, which is associated with NEC pathogenesis. EGF in AF mediates the dampening of TLR4 signaling *via* both the peroxisome proliferator-activated receptor and EGF receptor (23). *In vivo* experiments in Sprague-Dawley rats (born on day 21.5 of gestation, term gestation is 22.5–23 days) support the finding that AF has protective effects against NEC. Rats fed with formula supplemented with AF or formula supplemented with recombinant (r)HGF showed significantly reduced NEC frequency and severity as compared to the formula-fed rats. The protective effects of AF were attributed partly to the presence of HGF (24). Arguably, the dampening effect depends on the presence of multiple TFs and cannot be reduced to one single TF.

Consistent with these findings, NEC severity was reduced in preterm pigs (born on day 105–107 of gestation, term gestation is 114–118 days) fed with BM and porcine AF as compared to formula-fed pigs. AF-fed pigs had decreased intestinal bacterial colonization and lower expression of inflammatory genes (25). Another *in vivo* study compared NEC development in preterm pigs fed with parenteral nutrition and minimal enteral nutrition supplemented with porcine AF, HAF or a control. Increased body weight, reduced inflammatory response and reduced incidence of NEC were observed in pigs supplemented with porcine AF and HAF. In a follow-up experiment, pigs were fed similarly but after 2 days the feeding was followed by 2 extra days of enteral nutrition. Under these conditions, feeding with porcine AF or HAF did not protect against the development of NEC (26).

### Administration of Amniotic Fluid Stem Cells and Their Extracellular Vesicles

AFSCs have a phenotype in between embryonic stem cells and mesenchymal stem cells (MSCs). AFSCs express both pluripotency (e.g., octamer binding transcription factor-4, sex determining region Y-box 2, Rex1, cyclin A, Nanog) and mesenchymal markers [e.g., cluster of differentiation (CD)44, CD105, CD73, CD166, CD133, CD90]. AFSCs exhibit varying potential to differentiate into cell types of all germ layers. While embryonic stem cells are tumorigenic *in vivo*, studies in immune-compromised animals demonstrated that AFSCs are not (27). In contrast to bone marrow-derived MSCs (BM-MSCs), AFSCs can be isolated relatively easily (28). Together with the finding that AFSCs are easy to expand *in vitro*, AFSCs seem to be an interesting therapeutic candidate and are studied for tissue engineering in *in vitro* and *in vivo* models of disease (10). Various studies were conducted to identify the impact of AFSCs on the development of NEC (Supplementary Table 2).

Intraperitoneal injection of AFSCs had beneficial effects on Sprague-Dawley rats (born full term, term gestation is 22 days), compared to pups treated with phosphate buffer saline (PBS). AFSCs integrated in the intestinal walls and improved survival of NEC-induced rats. Gut damage was reduced while intestinal function was improved by increased cell proliferation and decreased apoptosis. AFSCs appeared to function through a different mechanism than BM-MSCs as the impact of AFSCs was, at least to some extent, mediated by modulation of cyclooxygenase-2-expressing stromal cells. AFSCs appeared to stimulate the release of specific GFs that act on intestinal progenitor cells, which can reduce inflammation and stimulate the formation of intestinal tissue (29). In prematurely delivered Lewis rats (born one half day premature, term gestation is 22 days), the effect of AF-MSCs and BM-MSCs on the development of NEC was compared. Both stem cells were associated with significantly lower NEC incidence and severity as compared to the breastfed control group (30). Another key study on intestinal organoids co-cultured with AFSCs as well as AFSC-administration in NEC-induced C57BL/6 mice (born full term, term gestation is 18.5 days) demonstrated that AFSCs prevented epithelial permeability and tight junction disruption through induction of a protective endoplasmatic reticulum stress response (31). Noteworthy, when full term C57BL/6 mice were treated prior to disease induction, AFSCs but not MSCs prevented injury. A possible explanation could be that the secreted protein panels are vastly different; proteins secreted from AFSCs function in cellular, developmental and metabolic processes while proteins secreted from MSCs play a role in immune processes (32).

Another approach uses extracellular vesicles (EVs) that have a similar content as the cells they are secreted by and are capable of affecting neighboring cells. EVs are divided in microvesicles (100–1,000 nm) and exosomes (30–150 nm). EVs contain cargo in the form of genetic material, mainly regulatory micro (miRNA) and bioactive factors (33). Using various techniques utilizing differential sedimentation, solubility and/or exclusion based on size, EVs with typical EV morphology and protein markers can be isolated from AFSCs (34). Moreover, Balbi et al. (35) illustrated that AFSCs secrete functional EVs that mediate processes of cellular proliferation, immunomodulation, anti-inflammation as well as exert pro-angiogenic and antiapoptotic effects.

Prematurely delivered Lewis rats were injected with exosomes derived from various stem cells, including AF-MSCs and BM-MSCs. Treatment with both types of exosomes reduced NEC incidence with similar effectivity as the stem cells they were derived from, supporting the potential for exosome-based therapy (36). In full term C57BL/6 mice, AFSC-derived EVs reduced NEC-induced intestinal injury by restoring epithelial regeneration and stimulating intestinal stem cells in a Wingless/Integrated-dependent manner. When AFSCs were injected prior to NEC onset, AFSCs were able to migrate and localize to the neonatal intestine and prevent NEC-induced injury (37). In line with this, mice treated with EVs derived from AFSCs showed reduced intestinal inflammation and injury while intestinal stem cell expression and cellular proliferation were enhanced (38). Noteworthy, the administration of conditioned medium (CM) derived from AFSCs was also studied in full term C57BL/6. Treatment with CM was associated with increased stem cell activity and recovery from NEC, similar to the effect of AFSCs-derived exosomes. While the composition of this CM differs from the physiological secretome as produced by AFSCs, AFSC-CM holds important cellular information (e.g., mRNA, miRNA, DNA, proteins, and EVs) (39).

### Postnatal Enteral Simulated Amniotic Fluid Administration

The final highlighted approach is enteral administration of simulated AF (SAF) (Supplementary Table 2). SAF is a sterile isotonic solution with a similar electrolyte composition to HAF with added bioactive factors as seen in AF. These bioactive factors include erythropoietin (EPO) and human granulocyte colony-stimulating factor (G-CSF), which play a role in intestinal repair and regeneration (40). Various feeding studies have been conducted in PIs and (V)LBWIs (Supplementary Table 2).

In a trial including 150 PIs (≤28 weeks) with low BW (≤1,250 g), one group received feedings according to a normal schedule, a second group received SAF without supplemented GFs and the last group received SAF with recombinant human (rh)EPO. The duration until full enteral feeding of infants fed with SAF or SAF with rhEPO was significantly decreased. This group also showed a significantly quicker weight gain and shorter hospital stay (41). While this study did not identify any impact of SAF on NEC development, another study demonstrated that enteral administration of rhEPO and rhG-CSF improved feeding outcomes and decreased the NEC risk in PIs (≤33 weeks) (42). This is in line with Khalesi et al. (43) and Wang et al. (44) who, respectively, demonstrated that enteral administration of rhG-CSF in VLBWIs (<1,500 g) was associated with a significantly lower NEC rate while administration of rhEPO in PIs (≤32 weeks) significantly reduced NEC incidence.

Another trial included 40 PIs/late term neonates who were recovering after GIT surgery. The treatment group received SAF with rhEPO and rhG-CSF. Feeding tolerance in this group improved more in comparison to the control. No conclusions could be drawn about the impact of SAF on NEC pathogenesis since there were no reported cases of NEC. Interestingly, this study found no differences in white blood cell count, hemoglobin and hematocrit levels between the treatment and control group, indicating that rhEPO and rhG-CSF did not have systemic impact (45). While EPO administration in PIs does not significantly increase the risk of retinopathy of prematurity, conflicting results on this topic call for cautiousness in clinical research (46). These studies showed that enteral feeding with SAF and/or supplemented GFs beneficially impacts feeding tolerance but the effect on the pathogenesis of NEC remains inconclusive. Further studies are necessary to adequately address the magnitude of SAF administration on the development of NEC.

## Challenges of Clinical Application of Amniotic Fluid-Based Therapy in the Context of Necrotizing Enterocolitis

**Figure 2:**
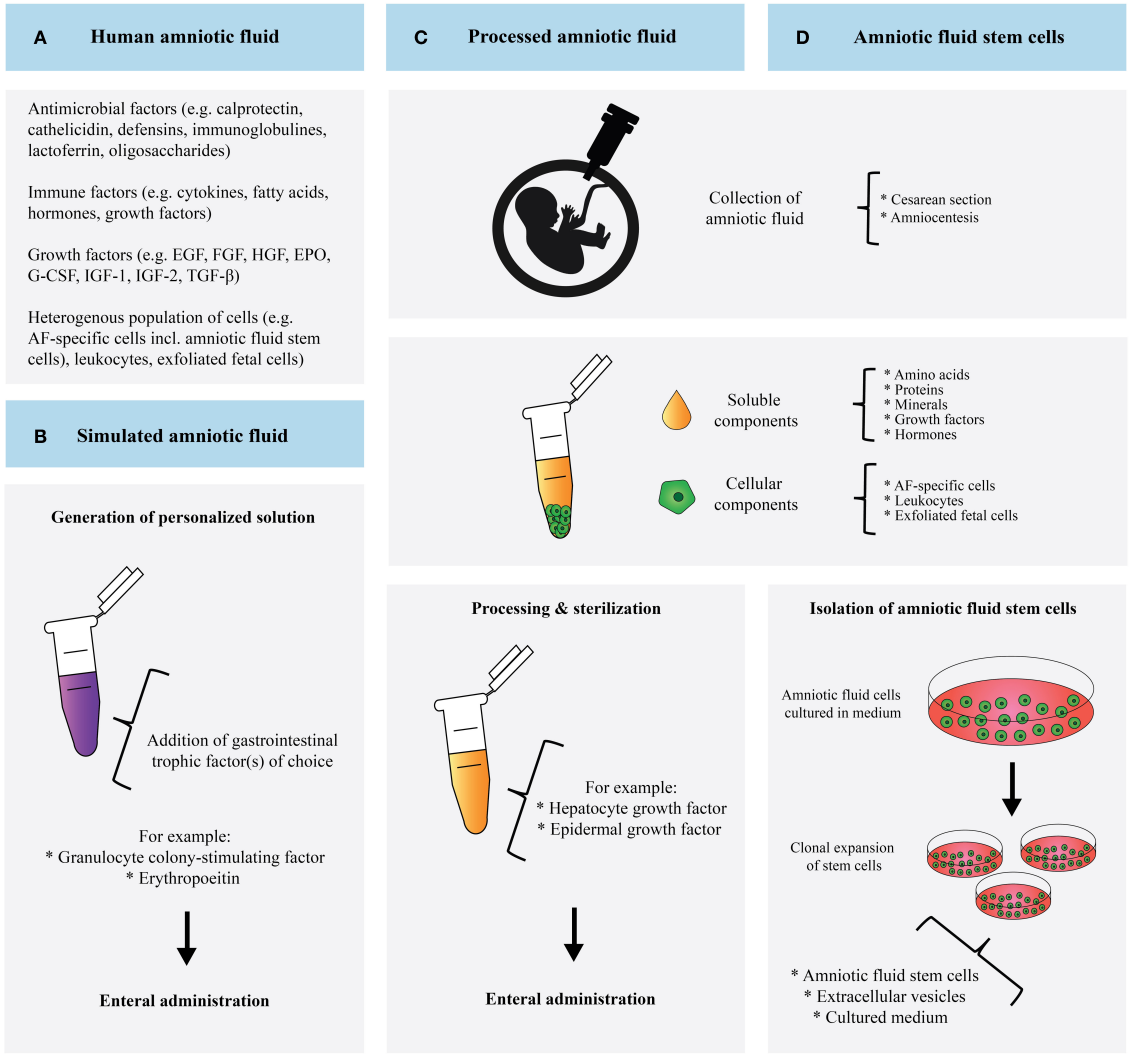
Schematic overview and comparison of human amniotic fluid **(A)**, simulated amniotic fluid **(B)**, processed amniotic fluid **(C)**, and amniotic fluid derived stem cells **(D)**.

Here, we have assessed the state of the art regarding the use of AF in the prevention and treatment of NEC in neonates (Figure 2). Although current literature lacks relevant clinical human data on this topic, we closely examined *in vitro* and *in vivo* studies as well as human studies on feeding tolerance, which have taken the first steps toward realizing the clinical application of AF-based therapy.

Each approach is accompanied by its own challenges. To be able to mimic AF swallowing by enteral PAF administration, high volumes of PAF must be supplemented. Fortunately, lyophilization is able to preserve TFs in HAF as well as improve cellular proliferation and reduce IL-8 production, supporting the notion that PAF administration may benefit vulnerable infants (47). Moreover, the PAF- and AFSCs-approach utilizes the body's own fluid in contrast to SAF. The PAF approach provides the benefits of all soluble components. Noteworthy, AF also contains components with antimicrobial and prebiotic properties that can potentially protect against NEC (13). The contribution of many of these molecules have yet to be identified. Since the cellular components of AF are removed during centrifugation, treatment with PAF excludes the protective effect of AFSCs. Vice versa, the administration of merely AFSCs lacks the beneficial effect of the soluble AF content.

Although SAF represents a carefully selected content of AF, it is likely that, unintentionally, equally important molecules are neglected in the formulation and the manufactured SAF might not represent AF accurately. A vast number of biologically important molecules have not been studied yet in the context of SAF. Therefore, our notion to explore large numbers of molecules expressed in AF and craft more complex SAF formulations is supported. Similar to PAF, SAF does not contain AFSCs and fails to incorporate their beneficial effect. While creating a personalized solution has advantages, it also makes resembling the *in utero* composition of AF more difficult. Significant research efforts are needed to characterize the functions of the single components in order to craft meaningful SAF. Noteworthy, SAF administration does not raise ethical issues compared to the use of HAF. In addition, issues how to effectively sterilize and store HAF for long-term use do not play a role in SAF production. Finally, careful consideration of the donor AF is required when choosing the PAF- or AFSC-approach. For the latter, specific AFSC characteristics should be taken into account. AFSCs derived during early second trimester are believed to have better potential than AFSCs derived from late second trimester. The more potent AFSCs have a smaller cell size, a more convenient cell density and a shorter dividing time; these characteristics are associated with more vigorous stem cell potential. AFSCs collected at an earlier stage of pregnancy may therefore have more therapeutic value than AFSCs derived from full-term pregnancies (48).

Of particular interest would be to investigate the potential effects of AF, AFSCs, and SAF on the intestinal microbiome. The preterm gut microbiome differs from the term microbiome, among other reasons, due to early initiation of enteral feeding. PIs often have higher numbers of facultative anaerobic bacteria, reduced levels of *Bifidobacterium* and *Bacteroides* and increased numbers of *Escherichia coli, Staphylococcus*, and *Klebsiella*, which can potentially be pathogenic. In addition to the role of TLR4 signaling, previous studies have demonstrated an association between the preterm gut microbiome and NEC (49). Various pre- and postnatal factors contribute to the development of this microbiome. The knowledge that the fetus ingests large volumes of AF during pregnancy and the finding that microbial DNA is present in meconium suggests that the fetal gut might be exposed to AF microbes *in utero*, although it is not yet known whether the fetal GIT is significantly affected by this (50). In addition, studies on BM, which has similar components to AF, have demonstrated that BM also modulates the neonatal microbiome (51). Future research should point out whether AF-based therapy results in alternation of the neonatal microbiome and what the subsequent effect would be on NEC development.

Although the research field of AF-based therapy has vastly expanded, key knowledge is still lacking to design and conduct a definitive randomized controlled trial. We argue that various steps need to be taken beforehand to fill in the gaps of knowledge in current literature. At this point research has focused on a variety of animal models, making it complicated to directly compare findings and translate the results to a human setting. Relevant preclinical human models that more closely resemble the immature intestine susceptible to NEC need to be studied to gain more knowledge on the effect of AF-based therapy in a clinical setting. The use of organoids from fetal intestines or induced pluripotent stem cells can provide insights on the effect of AF compounds on intestinal epithelial maturation, barrier function and innate immune response (52, 53). Moreover, further studies are needed to characterize the ideal dose and route of administration for the various approaches. Finally, research should focus on comparing the various AF-based approaches and determine which approach has most clinical potential in the context of NEC.

In conclusion, we have taken a close look at the current body of knowledge on the potential of AF-based approaches in the prevention and/or treatment of NEC. Future research is necessary to investigate whether these proposed approaches will benefit neonates susceptible to develop NEC and/or neonates suffering from NEC. We hope that this framework will help toward clinical application of AF for the treatment and/or prevention of NEC.

## Author Contributions

MvW, TdB, SS, and RdK were involved in the conception and design of the study. RdK drafted the manuscript. All authors revised the manuscript critically and read and approved the final manuscript.

## Conflict of Interest

The authors declare that the research was conducted in the absence of any commercial or financial relationships that could be construed as a potential conflict of interest.

## Publisher's Note

All claims expressed in this article are solely those of the authors and do not necessarily represent those of their affiliated organizations, or those of the publisher, the editors and the reviewers. Any product that may be evaluated in this article, or claim that may be made by its manufacturer, is not guaranteed or endorsed by the publisher.

## Abbreviations

AF: amniotic fluid
AFSC: amniotic fluid stem cell
BM: breast milk
BM-MSCs: bone marrow-derived mesenchymal stem cells
BW: birth weight
EGF: epidermal growth factor
EPO: erythropoietin
EV: extracellular vesicle
FGF: fibroblast growth factor
GA: gestational age
G-CSF: granulocyte-colony stimulating factor
GF: growth factor
GIT: gastrointestinal tract
HAF: human amniotic fluid
HGF: hepatocyte growth factor
IGF: insulin-like growth factor
IL: interleukin
LPS: lipopolysaccharide
NEC: necrotizing enterocolitis
MSC: mesenchymal stem cell
PAF: processed amniotic fluid
PBS: phosphate buffer solution
PI: preterm infant
rh: recombinant human
SAF: simulated amniotic fluid
TGF: transforming growth factor
TLR4: Toll-like receptor 4
TF: trophic factor
(V)LWBI: (very) low birth weight infant.
