# Supplementary material for: Amniotic Fluid: A Perspective on Promising Advances in the Prevention and Treatment of Necrotizing Enterocolitis
Source: Front Pediatr. 2022 Mar 14;10:859805. doi: 10.3389/fped.2022.859805 (PMC8964040; doi:10.3389/fped.2022.859805)
Supplement: Supplementary file 1 [file Table_1.pdf]

SUPPLEMENTARY MATERIAL

Supplementary table S1: Critical appraisal of referenced animal studies on processed amniotic fluid and amniotic fluid stem cells using SYRCLE's tool for assessing risk of bias. Per domain the risk of bias is described as 'high', 'unclear' or 'low' (Hooijmans et al., 2014).

**Type**	**Selection bias**			**Performance bias**		**Detection bias**		**Attrition bias**	**Reporting bias**	**Other**
**Domain**	[1] Sequence generation	Baseline characteristics	[3] Allocation concealment	[4] Random housing	[5] Blinding	[6] Random out-come assessment	[7] Blinding	[8] Incomplete outcome data	[9] Selective outcome reporting	[10] Other sources
**Approach 1:** processed amniotic fluid										
(23)	Unclear	Low	Unclear	Unclear	Unclear	Unclear	Low	Unclear	Low	Unclear
(24)	Unclear	Low	Unclear	Unclear	Unclear	Unclear	Low	Unclear	Low	Unclear
(25)	Low	Low	Unclear	Unclear	Unclear	Low	Low	Unclear	Low	Unclear
(26)	Low	Low	Unclear	Unclear	Unclear	Unclear	Unclear	Unclear	Low	Unclear
**Approach 2:** amniotic fluid stem cells										
(29)	Low	Low	Unclear	Unclear	Unclear	Unclear	Low	Unclear	Low	Unclear
(30)	Low	Low	Unclear	Unclear	Unclear	Unclear	Low	Low	Low	Unclear
(31)	Low	Low	Unclear	Unclear	Unclear	Unclear	Low	Unclear	Low	Unclear
(32)	Low	Low	Unclear	Unclear	Unclear	Unclear	Low	Low	Low	Unclear
(36)	Unclear	Low	Unclear	Unclear	Unclear	Unclear	Low	Low	Low	Unclear
(37)	Low	Low	Low	Unclear	Unclear	Unclear	Low	Low	Low	Unclear
(38)	Unclear	Low	Unclear	Unclear	Unclear	Unclear	Low	Low	Low	Unclear
(39)	Unclear	Low	Unclear	Unclear	Unclear	Unclear	Low	Unclear	Low	Unclear

Review authors judgement per domain: [1] Was the allocation sequence adequately generated and applied?; [2] Were the groups similar at baseline or were they adjusted for confounders in the analysis?; [4] Was the allocation adequately concealed?; [5] Were the animals randomly housed during the experiment? [6] Were the caregivers and/or investigators blinded from knowledge which intervention each animal received during the experiment?; [7] Were animals selected at random for outcome assessment?; [8] Were incomplete outcome data adequately addressed?; [9] Are reports of the study free of selective outcome reporting?; [10] Was the study apparently free of other problems that could result in high risk of bias?
