# Supplementary material for: Amniotic Fluid: A Perspective on Promising Advances in the Prevention and Treatment of Necrotizing Enterocolitis
Source: Front Pediatr. 2022 Mar 14;10:859805. doi: 10.3389/fped.2022.859805 (PMC8964040; doi:10.3389/fped.2022.859805)
Supplement: Supplementary file 2 [file Table_2.pdf]

frontiers

SUPPLEMENTARY MATERIAL

Supplementary table S2: Key studies which examine the impact of amniotic fluid-based therapies, including administration of processed amniotic fluid (A), administration of isolated amniotic fluid stem cells and its derivatives (B) and simulated amniotic fluid administration (C).

	Summary	Brief illustration of important findings
**Approach 1:** Processed amniotic fluid		
(23)	**Animal model:** C57BL/6 mice<br>**Design:** various experiments were conducted to determine the impact of amniotic fluid (AF) on Toll-like receptor 4 signaling in the fetal and neonatal intestinal epithelium, using e.g. epidermal growth factor receptor (EGFR) inhibitors.<br>**Conclusion:**<br>-Daily enteral AF administration decreased severity of necrotizing enterocolitis (NEC).<br>-*In vitro* experiments in intestinal epithelial cell line (IEC)-6 showed that the dampening effect on the Toll-like receptor 4 pathway is mediated by epidermal growth factor (EGFR) via peroxisome proliferator-activated receptor (PPAR-γ) and EGFR.	***In vitro* experiments in IEC-6:**<br>-Exposure to lipopolysaccharide (LPS) increased translocation of nuclear factor kappa B subunit 1 (NF-kB, transcription regulator) from cytoplasm to the nucleus at 1 hours and induced expression of interleukin (IL)-6 at 6 hours compared to IEC-6 not exposed to LPS (both p<0.05). Treatment with AF decreased both translocation of NF-kB and IL-6 expression when compared to the control group (both p<0.05).<br>-In IEC-6 exposed to LPS, EGF significantly reduced translocation of NF-kB and expression of IL-6 compared to the control group (both p<0.05). When EGFR was knocked out, using lentiviral transduction of EGFR short hairpin RNA, neither treatment with AF nor EGF was capable of reducing translocation of NF-kB or expression of IL-6.<br>-Similarly, when PPAR-γ was knocked out, using lentiviral transduction of PPAR-γ short hairpin RNA, neither treatment with AF nor EGF was capable of reducing translocation of NF-kB or expression of IL-6.<br>***In vivo* experiments on fetal and neonatal intestinal epithelium:**<br>-In NF-kB-green fluorescent protein reporter fetal mice, when LPS was delivered in the intestines together with murine AF, a significant increase in green fluorescent protein expression, inducible nitric oxide synthase (NOS) and IL-6 compared to mice injected with saline or AF alone was demonstrated (all p<0.05).<br>-While treatment of fetal mice with AF reduced IL-6 at 6 and 12 hours (both p<0.05) and inducible NOS at 6 and 12 hours (both p<0.05), administration of an EGFR inhibitor reversed these effects.<br>-In neonatal mice, LPS significantly reduced the expression of proliferation cell nuclear antigen (PCNA, p<0.05) as well as increased IL-6 at 6 and 12 hours (both p<0.05) and increased inducible NOS at 6 and 12 hours (both p<0.05), while administration of AF reversed these effects.<br>-Administration of EGFR inhibitor significantly diminished the effects on enterocyte proliferation and IL-6 expression when compared to the mice treated in the absence of the inhibitor (both p<0.05).<br>**Histological evaluation of intestinal tissue:**<br>-Compared to the NEC-induced newborn mice, AF significantly reduced NEC severity (p<0.05).<br>-Adding an EGFR inhibitor diminished these effects compared to the NEC-induced newborn mice (p<0.05).
(24)	**Animal model:** Sprague-Dawley rats<br>**Design:** *Experiment 1:* NEC-induced pups (n=80) were fed with formula or formula supplemented with 30% late-gestation rat AF; *Experiment 2:* NEC-induced pups (n=58) were fed with formula or formula supplemented with hepatocyte growth factor (HGF).<br>**Conclusion:**<br>-Supplementation with AF or HGF showed reduced NEC frequency and severity; HGF is partly responsible for the	**Tissue collection and NEC evaluation:**<br>-NEC severity was reduced in the intervention group treated with rat AF compared to the control group (median injury grade 0 in the intervention group vs. median injury grade 2 in the control group, p<0.01).<br>-Incidence of NEC-like injury was also reduced in the intervention group treated with rat AF compared to the control group (63% in control group vs. 41% in the AF groups, p<0.01).<br>**Cytokine expression patterns:**<br>-HGF was the most abundant cytokine in rat AF (n=5, collected at day 17-18, median 4,892 pg/ml) and human AF (n=30, collected at ≥39 weeks of gestation, median 8,354 pg/ml).<br>***In vitro* experiments in IEC-6:**<br>-*Proliferation*: determination of bromodeoxyuridine incorporation in IEC-6 demonstrated that treatment with 10%

	protective effect of AF.<br>-*In vitro* experiments in IEC-6 showed that HGF increased epithelial cell migration, proliferation and survival.	and 30% AF increased the optical density compared to the control group (respectively, p<0.05 and p<0.001). Similarly, treatment with HGF as well as HGF and AF also increased the optical density (both p<0.05).<br>-*Migration:* treatment with 10% and 30% AF increased the number of migrating cells compared to the control group (respectively, p<0.05 and p<001). Similarly, treatment with HGF as well as HGF and AF had the same effect compared to the control group (both p<0.05).<br>-*Survival*: an XTT survival assay in the presence of cycloheximide and tumor necrosis factor (TNF) demonstrated that treatment with 10% and 30% AF increased the optimal density compared to the control group (both p<0.05). Similarly, treatment with HGF also increased the optical density (p<0.05).<br>-The effect of AF on proliferation, migration and survival were reversed in the presence of anti-HGF antibody.<br>**Enteral administration of HGF in animal model:**<br>-NEC severity was reduced in the intervention group treated with formula and HGF compared to the control group treated with formula alone (p<0.01). Similarly, NEC incidence was significantly reduced (p<0.05).
(25)	**Animal model:** preterm pigs<br>**Design:** *Experiment 1:* preterm pigs were fed with parenteral nutrition for two days followed by two days of enteral feeding with porcine colostrum (n=7), formula (n=13) or AF before and after formula introduction (n=10); *Experiment 2*: similar to experiment 1 but suppletion only *during* formula feeding.<br>**Conclusion:**<br>-Supplementation of AF prior to full enteral nutrition resulted in decreased intestinal bacterial populations and decreased expression of inflammatory genes.<br>-The reduction of the NEC score was similar in pigs fed with colostrum compared to pigs fed with AF.	**NEC evaluation and tissue collection:**<br>-When AF was supplemented prior to full enteral feeding, the NEC score was significantly lower in the AF and colostrum group compared to the formula-fed group (respectively, 9.9 and 7.7 vs. 17.3, p<0.05).<br>-There was no effect of AF administration when *only* given during enteral feeding.<br>**Intestinal microbiology:**<br>-Hybridization with an eubacterial probe demonstrated that bacterial colonization was lowest in the group given AF prior to full enteral feeding, intermediate in the formula-fed group and highest in the colostrum group.<br>**Microarray analysis, quantitative real-time PCR:**<br>-When comparing the AF group to the formula-fed group *with* NEC, genes that play a role in the innate immune response (e.g. IL-1α, NOS2, TNF-α were downregulated, p<0.01).<br>-When comparing the AF group to the formula-fed group *without* NEC, 79% of the 24 genes that were differentially regulated in the AF group differed from the differentially regulated genes in the formula-fed group.<br>-*In the middle small intestine:* when comparing the AF group to the formula-fed group with NEC several pro-inflammatory cytokines (e.g. IL-1α, TNF-α and IL-6) and the neutrophil attractant IL-8 were downregulated (all p<0.05); *In the distal small intestine:* when comparing the AF group to the formula-fed group with NEC, TNF-α and IL-1α were significantly downregulated (both p<0.05).
(26)	**Animal model:** preterm pigs<br>**Design:** *Experiment 1*: neonatal pigs were fed with parenteral nutrition and minimal enteral nutrition with porcine AF, human AF or a control fluid (all n=9) for two days; *Experiment 2*: after the feeding schedule of experiment 1, all pigs were fed for two days with enteral nutrition (all n=10-12).<br>**Conclusion:**<br>-Supplementation with porcine and human AF improved weight gain and reduced the inflammatory response during parenteral nutrition but did not protect against NEC later on.<br>-*In vitro* experiments in IEC-6 showed that porcine and human AF stimulated enterocyte proliferation/migration.	**NEC evaluation and tissue collection:**<br>-*Experiment 1:* after two days, there were no significant differences in NEC incidence among the treatment groups but increased body weight gain was seen in the porcine AF and human AF group compared to the control group (40.9 ± 3.0 for porcine AF and 52.0 ± 3.0 for human AF vs. 18.6 ± 3.0 g/kg/d for the control group, p<0.01).<br>-*Experiment 2*: NEC incidence was significantly higher in the human AF group compared to the control group (9/10 pigs vs. 4/11 pigs, p<0.05), while the incidence was similar in the porcine AF and the control group (6/10 pigs vs. 4/11 pigs, p=n.s.). Increased body weight gain was seen in both the porcine and human AF group compared to the control group (8.2 ± 2.4 and 11.5 ± 2.6 vs. −2.2 ± 2.4 g/kg/d; p<0.001).<br>**IL-8 and IL-6 content in the intestines:**<br>-*Experiment 1:* significantly increased IL-8 and IL-6 were seen in the control group compared to the human AF group (respectively, 1.4-fold increase, p<0.05, and 2.5-fold increase, p<0.01). Similarly, IL-6 was significantly increased in the control group compared to the porcine AF group (2.0-fold increase, p<0.01).<br>-*Experiment 2*: significantly increased IL-6 and IL-8 were seen in the AF group compared to the control group (for IL-6 2.5-fold increase and for IL-8 4.5-fold increase, both p<0.05). Similarly, IL-6 was significantly increased in the porcine AF group when compared to the control group (2.5-fold increase, p<0.05).

		***In vitro* experiments on IEC-6:**<br>-Porcine and human AF stimulated enterocyte proliferation and migration compared to the control group (for proliferation 1.3-fold increase and 1.5-fold increase, both p<0.001; for migration both 2.5-fold increase, p<0.001).
**Approach 2:** Isolated amniotic fluid stem cells		
(29)	**Animal model:** Sprague-Dawley rats<br>**Design:** *Experiment 1:* NEC-induced pups were divided in groups based on the treatment: amniotic fluid stem cells (AFSCs) (n=40), bone-marrow derived-mesenchymal stem cells (BM-MSCs) (n=17) and phosphate buffered saline (PBS) (n=24). The control group consisted of breastfed (BF) rats (n=10); *Experiment 2*: NEC-induced (n=77) and BF (n=32) pups were divided in subgroups based on the treatment: dimethyl sulfoxide (DMSO), cyclooxygenase (COX)-2 inhibitor, COX-1 and -2 inhibitor or COX-2 inhibitor.<br>**Conclusion:**<br>-AFSCs improved survival of NEC, decreased NEC incidence and damage to the gut, improved function of the intestines, increased enterocyte proliferation and reduced apoptosis.<br>-The beneficial effect was attributed to modulation of stromal cells expressing COX-2 in the lamina propria of the intestines.	**Survival curve, imaging, gut function:**<br>-The AFSC group demonstrated improved survival compared to the BM-MSC (p=0.024) or PBS group (p<0.0001).<br>-The PBS group demonstrated dilated bowel loops with thinned gut walls compared to the AFSC group.<br>-*Carmine red solution:* intestinal motility was similar in the AFSC group compared to the BF group (p=n.s.), while motility was decreased in the PBS group compared to the BF group (p<0.001).<br>-*Plasma lactulose/mannitol ratio:* the AFSC group demonstrated decreased intestinal permeability compared to the PBS group (0.031±0.004, p<0.005).<br>**Gene expression (cDNA microarray):**<br>-Proteins involved in inflammation, tissue repair and enterocyte differentiation divided the AFSC from PBS group.<br>**Gut inflammation, apoptotic index, enterocyte migration/proliferation:**<br>-For the AFSC group, the malondialdehyde level (marker for oxidative stress) was not significantly reduced compared to the PBS group. Similarly, myeloperoxidase activity (marker for neutrophil infiltration) and cleaved caspase 3 (marker for epithelial apoptosis) positive cells/crypt were decreased (both p<0.05).<br>-For the AFSC group, the enterocyte migration/proliferation index as determined by 5-ethynyl-2'-deoxyuridine was increased compared to the PBS group (75% in AFSC group vs. 53% in the PBS group, p<0.0001).<br>**COX inhibitors survival study:**<br>-Clinical sickness score in the AFSC group treated with COX-2- or COX-1 and -2 inhibitors compared to the DMSO group was decreased (respectively, p<0.0001 and p<0.01) but was preserved when treated with COX-1 inhibitors.
(30)	**Animal model:** Sprague-Dawley rats<br>**Design:** NEC-induced pups were intraperitoneally injected with PBS (n=62) or stem cells, e.g. AF-MSCs (n=42) and BM-MSCs (n=48). The control group was BF (n=10).<br>**Conclusion:**<br>Administration of AF-MSCs and BM-MSCs reduced experimental NEC incidence and severity.	**Histologic grading of intestinal tissue:**<br>-Treatment with AF-MSCs reduced the NEC incidence from 61.3% to 19.1% (n=42, p<0.0001), while treatment with BM-MSCs reduced the NEC incidence to 22.9% (n=48, p<0.0001).<br>-In terms of severe NEC incidence (regarded as grade 3 or grade 4), when treated with AF-MSCs, 14% had grade 3 compared to 18% grade 3 and 16% grade 4 in the PBS group (p=0.025). When treated with BM-MSCs, 4% had grade 3 compared to the 18% grade 3 and 16% grade 4 in the PBS group (p<0.0001).
(31)	**Animal model:** C57BL/6 mice<br>**Design:** *Experiment 1:* NEC-induced intestinal organoids from the small intestine of neonatal mice were cultured with PBS or AFSCs; *Experiment 2*: following NEC induction on postnatal day 5, pups were intraperitoneally injected with PBS (n=10) or AFSCs (n=10) on postnatal day 6 and 7. The control group consisted of BF mice (n=10).<br>**Conclusion:**<br>-Administration of AFSCs prevented epithelial permeability and tight junction disruption in intestinal organoids.<br>-AFSCs also restored tight junction activity and rescued intestinal permeability in NEC pups.	**Organoids (permeability, tight junctions):**<br>-Fluorescein isothiocyanate-dextran (FD4, marker for intestinal permeability) was decreased in organoids treated with AFSCs compared to PBS (p<0.05).<br>-Zonula occludens-1 staining (tight junction marker) was expressed in the form of a ring along the apical intestinal epithelial membrane, which was disrupted by NEC. When the organoids were treated with AFSCs this pattern was reversed. Similarly, NEC induction significantly upregulated expression of the gene for the Claudin 2 protein (tight junction marker) and downregulated the gene for the Claudin 7 protein (tight junction marker) as compared to PBS (both p<0.05); when the organoids were treated with AFSCs this was reversed.<br>**Organoids ER stress pathway:**<br>-Gene expression involved in the ER stress pathway (e.g. *ATF6, IRE1, PERK, ATF4, CHOP)* was increased in organoids treated with AFSCs compared to PBS (respectively, p<0.01, p<0.01, p<0.01, p<0.001 and p<0.001).<br>-HA15, an inhibitor of a downstream effector of the ER stress pathway, diminished the upregulation of these

	-The protective endoplasmatic reticulum (ER) stress response restored tight junction activity and rescued permeability.	effectors (for *ATF6* p<0.01, for *IRE1* p<0.01, for *PERK* p<0.0, for *ATF4* p<0.001 and for *CHOP* p<0.001) when compared to the AFSC group as well as averted the changes in zonula occludens-1, Claudin 2 and Claudin 7.<br>**Histological grading of intestinal tissue:**<br>-Histological grading was significantly lower in the AFSC group compared to the PBS group (p<0.001).<br>**Gene quantification, immunostaining of terminal ileum:**<br>-Cleaved caspase 3 staining was significantly decreased in the AFSC group compared to PBS group (p<0.01).<br>-Similar to organoids, the expression of BiP and CHOP (e.g. proteins involved in the ER stress pathway) was increased in the AFSC group compared to the PBS group (both p<0.01).<br>-Claudin 7 gene and protein expression were significantly increased in the AFSC group compared to the PBS group (respectively, p<0.05 and p<0.001); Claudin 2 gene and protein expression were significantly decreased in the AFSC group compared to the PBS group (respectively, p<0.01 and p<0.001).
(32)	**Animal model:** C57BL/6 mice<br>**Design:** NEC was induced in 5-day-old mice. Prior to disease induction, mice pups were intraperitoneally injected with PBS (n=10) or rat stem cells: AFSCs (n=10) or MSCs (n=10). The control group were BF mice (n=10).<br>**Conclusion:**<br>-Administration of AFSCs was associated with increased intestinal growth and decreased NEC-induced injury.<br>-This was in contrast with administration of MSCs which were not capable of preventing intestinal injury.<br>-Distinct protein secretory profiles were demonstrated in the supernatant of AFSCs and MSCs.	**Histologic grading of intestinal issue**:<br>-In the PBS and MSC group, NEC developed in respectively 7/10 pups and 6/10 pups compared to the AFSC group, where 0/10 pups developed NEC-like injury (both p<0.0001).<br>**Gene quantification, immunostaining of terminal ileum:**<br>-Gene expression of IL-6 was significantly reduced in the AFSC group compared to the PBS group (p<0.001), while IL-6 was not significantly reduced in the MSC group (p=n.s.).<br>-Ki67 (marker of proliferation) and leucine-rich repeat-containing G-protein coupled receptor *(*Lgr5, intestinal stem cell marker) were elevated in the AFSC group compared to the PBS group (both p<0.001), while in the MSC group Ki67 and Lgr5 were not significantly elevated (p=n.s.).<br>**Proteomic analysis:**<br>-Gene ontology demonstrated that AFSC-secreted proteins were involved in 'biological adhesion, cellular processes, development, growth, metabolism and reproduction', while MSC-secreted proteins were involved immune-system related processes; both secretomes were involved in regulation of cell growth and cell size.
(36)	**Animal model:** Lewis rats<br>**Design:** NEC-induced rat were intraperitoneally injected with PBS (n=28) or exosomes derived from stem cells, including AF-MSCs (n=38) and BM-MSCs (n=28). The control group consisted of BF mice (n=10).<br>**Conclusion:**<br>Administration of exosomes isolated from AF-MSCs and BM-MSCs reduced NEC incidence and severity.	**Histologic grading of intestinal tissue:**<br>-NEC incidence was reduced to 25.0% in the group treated with exosomes derived from AF-MSCs and 23.1% when treated with exosomes derived from BM-MSCs (both p<0.05).<br>-When treated with exosomes from AF-MSCs, 15% had grade 4 NEC, 5.0% had grade 3 and 5.0% had grade 2 compared to 14.3% grade 4, 25% grade 3 and 21.4% grade 2 in the PBS group (p<0.05). When treated with BM-MSCs, 23.1% had grade 2 compared to the 14.3% grade 4, 25% grade 3 and 21.4% grade 2 in the PBS group (p<0.05).
(37)	**Animal model:** C57BL/6 mice<br>**Design:** EVs were harvested from AFSC medium. *Experiment 1:* NEC was induced on postnatal day 5 until 9 in neonatal mice, followed by intraperitoneal injection on postnatal day 6 with PBS (n=16), AFSCs (n=10) or Wingless related integration site (Wnt)-deficient AFSCs (n=8). BF mice consisted of the control group (n=10); *Experiment 2*: neonatal mice received AFSC-derived extracellular vesicles (EVs) and AFSCs (n=8); *Experiment 3*: prior to NEC induction neonatal mice received PBS (n=8) or AFSC-EV (n=8).	**Intestinal morphology analysis, immunostaining, gene expression:**<br>-*Experiment 1:* NEC grades were decreased in the AFSC group compared to the NEC-PBS group (p<0.01).<br>-Ki67 and Lgr5 were increased in the AFSC group compared to the NEC-PBS group (both p<0.001).<br>-*Experiment 1:* Compared to the control group, administration of Wnt-producing AFSCs was associated with improved epithelial morphology and decreased NEC grades (p<0.001), reduced expression of IL-6 and TNF-α (both p<0.001) as well as increased Ki67 and increased gene expression of *Lgr5* (respectively, p<0.01 and p<0.001). In the presence of Wnt-inhibitors, these findings were not observed.<br>-*Experiment 2*: Compared to the control group, AFSC-EVs were associated with improved epithelial morphology, decreased NEC grades (p<0.05), reduced IL-6 and TNF-α expression (both p<0.001) as well as increased Ki67 and increased gene expression of *Lgr5* (respectively, p<0.001 and p<0.01).

	**Conclusion:**<br>AFSCs and AFSC-EVs dampened intestinal injury by restoring epithelial regeneration as well as stimulating intestinal stem cells in a Wnt-dependent manner.	-*Experiment 3:* Compared to the control group, administration of AFSC-EVs prior to NEC induction was associated with increased epithelial proliferation but not reduced intestinal injury.<br>***In vitro* experiments in organoids:**<br>-Wnt activity was reduced in AFSCs treated with Wnt inhibitors compared to the control (for both inhibitors $p<0.05$).<br>-Compared to organoids co-cultured with AFSCs, intestinal organoids co-cultured with Wnt-deficient-AFSCs showed decreased number of organoids (for both inhibitors $p<0.01$) and surface area (for both inhibitors $p<0.01$).<br>-When AFSC-EVs were added to organoids, compared to the control, the round organoid percentage was increased ($p<0.01$) as well as the surface area ($p<0.001$), similar to addition of Wnt alone (respectively, $p<0.01$ and $p<0.001$).
(38)	**Animal model:** C57BL/6 mice<br>**Design:** EVs were derived from AFSCs. 5-day old pups were induced with NEC on postnatal day 5 until 9 followed by intraperitoneal injection with PBS (n=10) or EVs (n=11) on day postnatal day 6 and 7. The control group was BF (n=11).<br>**Conclusion:**<br>NEC incidence, intestinal injury and intestinal inflammation were reduced when treated with AFSC-EVs, while intestinal stem cell expression and cellular proliferation were enhanced.	**Histologic grading of intestinal issue**:<br>-In the PBS group, 80% had a histological score >2 compared to 18% in EV group.<br>-The EV group had a lower intestinal injury compared to the PBS group ($p=0.0048$).<br>**Gene quantification, immunostaining of intestinal tissue:**<br>-IL-6 and TNF-α expression were lower in the EV group compared to the PBS group (both $p<0.0001$).<br>-Lgr5 expression was increased in the EV group compared to the PBS group ($p=0.0003$).<br>-Ki67 expression was increased for the EV group compared to the PBS group ($p<0.0001$).
(39)	**Animal model:** C57BL/6 mice<br>**Design:** conditioned medium (CM) was derived from AFSCs. Mice were induced with NEC on postnatal day 5 until 9 followed by intraperitoneal injection with PBS (n=20) or human AFSC-CM (n=14) on postnatal day 6 and 7. The control group was BF (n=10).<br>**Conclusion:**<br>-Administration of CM reduced intestinal damage as well as decreased mucosal inflammation, decreased infiltration of neutrophils, decreased epithelial apoptosis and improved intestinal angiogenesis.<br>-NEC-induced pups treated with CM were associated with restored intestinal regeneration and recovery.<br>-Peptides involved in regulation of immune and stem cell activity were identified in CM.	**Survival curve, histologic grading of intestinal issue:**<br>-Although there was at trend that treatment with CM improved survival of NEC-induced pups, there was no significant difference in terms of survival when compared to the NEC-PBS group.<br>-In 20% of the CM group NEC-like injury was demonstrated compared to 89% in the NEC-PBS group (p=n.s.).<br>**Gene quantification, immunostaining of intestinal tissue:**<br>-IL-6 and TNF-α were significantly reduced in the CM group compared to the PBS group (both $p<0.001$).<br>-Ki67 and Lgr5 were elevated in the CM group compared to the PBS group ($p<0.001$, $p<0.05$ and $p<0.001$).<br>-Myeloperoxidase positive cells/crypt were reduced in the CM group compared to the PBS group ($p<0.001$).<br>-Cleaved caspase 3 positive cells/crypt were decreased and vascular endothelial growth factor was increased (respectively, $p<0.001$ and $p<0.05$).<br>**Proteomic analysis:**<br>-Proteins that play a role in immune-regulation, cell cycle and stem cell regulation were identified in CM.
**Approach 3:** Simulated amniotic fluid		
(41)	**Design:** *Group 1*: preterm infants (PIs) (<28 weeks and birth weight <1250 grams) with routine feeding schedule (n=50); *group 2*: PIs received simulated amniotic fluid (SAF, n=50); *group 3*: PIs received SAF and recombinant human erythropoietin (rhEPO) (n=50). Feedings started on postnatal day 3 and continued for 21 days.	**Evaluation of clinical and nutritional data:**<br>-The mortality rate in PIs receiving SAF or SAF-rhEPO was significantly lower than in the control group (4% in the SAF group and 3% in the SAF-rhEPO group vs. 16% in the control group, $p=0.027$).<br>-Frequency of complications were not significantly different; there were no significant differences in NEC frequency among the groups (6% in the SAF and 6% in the SAF-rhEPO group vs. 8% in the control group, $p=0.841$).

	**Conclusion:**<br>-Mortality rates in both AF and SAF-rhEPO group were significantly lower than the control group.<br>- NEC frequency or other complications did not significantly differ among the groups.	
(42)	**Design:** *Group 1*: PIs (<33 weeks) that received water (n=30); *group 2:* received recombinant human granulocyte-colony stimulating factor (G-CSF) (n=20); *group 3:* PIs that received rhEPO (n=20); *group 4:* PIs that received both rhG-CSF and rhEPO (n=20). Feeding started at the start of enteral nutrition for maximum of 7 days or was discontinued earlier if full enteral nutrition was reached.<br>**Conclusion:**<br>-Feeding tolerance was better in all treatment groups.<br>-Quicker weight gain and shorter hospital stay were significant in the treatment groups compared to the control group, in addition NEC risk was reduced from 10% to 0%.	**Evaluation of clinical and nutritional data:**<br>-When comparing the control group to the rhG-CSF, rhEPO and rhG-CSF+rhEPO group, there was a shorter time to achieve half and full enteral feeding (all p<0.05).<br>-Duration of withholding feeding was shorter in rhG-CSF+rhEPO group (p=0.037).<br>-Weight gain was quicker and hospital stay was shorter in the groups treated with rhG-CSF, rhEPO and rhG-CSF+rhEPO compared to the control group (respectively, p=0.002, p=0.016 and p=0.004 for weight gain and p<0.001 for all groups for duration hospital stay).<br>-Significant differences in hemoglobin level, white blood cell level and platelet counts were lacking between the control and the treatment groups (p=n.s.). In addition, serum levels of G-CSF and EPO at 0 and 7 days after commencing treatment did not significantly differ across the treatment groups (p=n.s.)
(43)	**Design:** The treatment group consisted of VLBWIs (<1500 grams) that received enteral administration of G-CSF for 7 consecutive days (n=81), while the control group consisted of infants admitted prior that did not receive G-CSF (n=191).<br>**Conclusion:**<br>Neonates that received G-CSF showed better feeding tolerance and a lower NEC incidence.	**Evaluation of clinical and nutritional data:**<br>-When comparing the control group to the G-CSF group, there was a shorter time to achieve 50, 75, 100 and full enteral feeding of 150 ml/kg/day (p<0.05).<br>-The rate of NEC was lower in the treatment group when compared to the control group (3.7% in the treatment group vs. 21% in the control group, p<0.005).
(44)	**Design:** 1285 PIs (<32 weeks) were randomized to receive rhEPO every other day for two weeks or a control fluid and were analyzed at 36 weeks of corrected age for NEC incidence.<br>**Conclusion:**<br>Treatment with rhEPO reduced the incidence of NEC.	**Evaluation of NEC incidence and severity:**<br>-The treatment group had a significantly lower incidence of NEC (12% for the treatment group vs. 17.1% for the control group, p=0.010). Specifically, for stage II and II incidence was decreased (3.0% for the treatment group vs. 5.4% for the control group, p=0.027).
(45)	**Design**: 40 late preterm/term neonates, in recovery from GIT surgeries, were divided in two groups. The treatment group received SAF with rhG-CSF and rhEPO (n=20), while the control group received distilled water (n=20).<br>**Conclusion:**<br>rhG-CSF and rhEPO improved feeding tolerance compared to the control group, while there were no reported NEC cases.	**Evaluation of clinical and nutritional data:**<br>-The SAF group achieved 50, 100, 120 mL and full enteral feeds earlier compared to the control group (respectively, p=0.047, p=0.032, p=0.041 and p=0.048). Similarly, this group also discontinued total parenteral nutrition earlier and had a higher weight gain rate (respectively, p=0.042 and p=0.033).<br>-There were no NEC cases observed but the SAF group had a lower incidence of abdominal distension (p=0.011).

Abbreviations: AF, amniotic fluid; AFSC, amniotic fluid stem cell; BM-MSC, bone-marrow derived-mesenchymal stem cell; COX, cyclooxygenase; DMSO, dimethyl sulfoxide; EGF, epidermal growth factor; EGFR, epidermal growth factor receptor; ER, endoplasmatic reticulum; EV, extracellular vesicles; IL, interleukin; HGF, hepatocyte growth factor; IEC, intestinal epithelial cell line; Ki67, marker of proliferation; Lgr5, intestinal stem cell marker; NOS, nitric oxide synthase; NF-kB, nuclear factor kappa B; PPAR-γ, peroxisome proliferator-activated receptor; PI, preterm infant; PBS, phosphate buffered saline; PCNA, proliferation cell nuclear antigen; rhEPO, recombinant human erythropoietin; rhG-CSF, recombinant human granulocyte-colony stimulating factor; SAF, simulated amniotic fluid; TNF, tumor necrosis factor; Wnt, wingless related integration site.
